# Findings from the Tushirikiane mobile health (mHealth) HIV self‐testing pragmatic trial with refugee adolescents and youth living in informal settlements in Kampala, Uganda

**DOI:** 10.1002/jia2.26185

**Published:** 2023-10-18

**Authors:** Carmen H. Logie, Moses Okumu, Isha Berry, Robert Hakiza, Stefan D. Baral, Daniel Kibuuka Musoke, Aidah Nakitende, Simon Mwima, Peter Kyambadde, Miranda Loutet, Shamilah Batte, Richard Lester, Stella Neema, Katie Newby, Lawrence Mbuagbaw

**Affiliations:** ^1^ Factor‐Inwentash Faculty of Social Work University of Toronto Toronto Ontario Canada; ^2^ Women's College Research Institute Women's College Hospital Toronto Ontario Canada; ^3^ United Nations University Institute for Water, Environment & Health Hamilton Ontario Canada; ^4^ Centre for Gender & Sexual Health Equity Vancouver British Columbia Canada; ^5^ School of Social Work University of Illinois Urbana‐Champaign Urbana Illinois USA; ^6^ School of Social Sciences Uganda Christian University Mukono Uganda; ^7^ Dalla Lana School of Public Health University of Toronto Toronto Ontario Canada; ^8^ Young African Refugees for Integral Development (YARID) Kampala Uganda; ^9^ Johns Hopkins Bloomberg School of Public Health Johns Hopkins University Baltimore Maryland USA; ^10^ International Research Consortium (IRC) Kampala Uganda; ^11^ National AIDS and STI Control Programme, Ministry of Health Kampala Uganda; ^12^ Most at Risk Population Initiative Mulago Hospital Kampala Uganda; ^13^ Organization for Gender Empowerment and Rights Advocacy (OGERA Uganda) Kampala Uganda; ^14^ Department of Medicine University of British Columbia Vancouver British Columbia Canada; ^15^ Department of Sociology and Anthropology Makerere University Kampala Uganda; ^16^ Centre for Research in Psychology and Sport Sciences School of Life and Medical Sciences University of Hertfordshire Hatfield UK; ^17^ Department of Health Research Methods, Evidence and Impact McMaster University Hamilton Ontario Canada; ^18^ Department of Anesthesia McMaster University Hamilton Ontario Canada; ^19^ Department of Pediatrics McMaster University Hamilton Ontario Canada; ^20^ Biostatistics Unit, Father Sean O'Sullivan Research Centre St Joseph's Healthcare Hamilton Ontario Canada; ^21^ Centre for Development of Best Practices in Health (CDBPH) Yaoundé Central Hospital Yaoundé Cameroon; ^22^ Division of Epidemiology and Biostatistics Department of Global Health Stellenbosch University Cape Town South Africa

**Keywords:** HIV self‐testing, refugees, youth, Uganda, humanitarian health, mHealth

## Abstract

**Introduction:**

Urban refugee youth remain underserved by current HIV prevention strategies, including HIV self‐testing (HIVST). Examining HIVST feasibility with refugees can inform tailored HIV testing strategies. We examined if HIVST and mobile health (mHealth) delivery approaches could increase HIV testing uptake and HIV status knowledge among refugee youth in Kampala, Uganda.

**Methods:**

We conducted a three‐arm pragmatic controlled trial across five informal settlements grouped into three sites in Kampala from 2020 to 2021 with peer‐recruited refugee youth aged 16–24 years. The intervention was HIVST and HIVST + mHealth (HIVST with bidirectional SMS), compared with standard of care (SOC). Primary outcomes were self‐reported HIV testing uptake and correct status knowledge verified by point‐of‐care testing. Some secondary outcomes included: depression, HIV‐related stigma, and adolescent sexual and reproductive health (SRH) stigma at three time points (baseline [T0], 8 months [T1] and 12 months [T2]). We used generalized estimating equation regression models to estimate crude and adjusted odds ratios comparing arms over time, adjusting for age, gender and baseline imbalances. We assessed study pragmatism across PRECIS‐2 dimensions.

**Results:**

We enrolled 450 participants (50.7% cisgender men, 48.7% cisgender women, 0.7% transgender women; mean age: 20.0, standard deviation: 2.4) across three sites. Self‐reported HIV testing uptake increased significantly from T0 to T1 in intervention arms: HIVST arm: (27.6% [*n* = 43] at T0 vs. 91.2% [*n* = 135] at T1; HIVST + mHealth: 30.9% [*n* = 47] at T0 vs. 94.2% [*n* = 113] at T1]) compared with SOC (35.5% [*n* = 50] at T0 vs. 24.8% [ = 27] at T1) and remained significantly higher than SOC at T2 (*p*<0.001). HIV status knowledge in intervention arms (HIVST arm: 100% [*n* = 121], HIVST + mHealth arm: 97.9% [*n* = 95]) was significantly higher than SOC (61.5% [*n* = 59]) at T2. There were modest changes in secondary outcomes in intervention arms, including decreased depression alongside increased HIV‐related stigma and adolescent SRH stigma. The trial employed both pragmatic (eligibility criteria, setting, organization, outcome, analysis) and explanatory approaches (recruitment path, flexibility of delivery flexibility, adherence flexibility, follow‐up).

**Conclusions:**

Offering HIVST is a promising approach to increase HIV testing uptake among urban refugee youth in Kampala. We share lessons learned to inform future youth‐focused HIVST trials in urban humanitarian settings.

## 1 INTRODUCTION

Urban refugee youth are understudied in HIV testing research. In fact, forcibly displaced people are described as “left behind on the path to 90‐90‐90” [1]. This knowledge gap is notable given that refugee youth often experience combinations of poverty, violence and disrupted sexual health services that enhance HIV vulnerabilities [2, 3, 4]. As reported across systematic reviews [5, 6, 7], HIV self‐testing (HIVST) is a promising approach for increasing HIV testing access and uptake by enhancing convenience and privacy. It is particularly important to examine HIVST feasibility and acceptability with refugee youth who may experience intersecting stigma, including HIV‐related stigma, refugee stigma and gender discrimination [8, 9, 10], as well as logistical barriers (e.g. language and transport costs) [10, 11, 12, 13]. Nonetheless, HIVST remains understudied with refugee adults and youth [14] despite its established ability to mitigate HIV testing barriers [5, 6, 7], including with youth in sub‐Saharan Africa (SSA) [15, 16, 17].

As the SSA nation hosting the most refugees (>1.4 million) [18, 19], Uganda is an exemplary context for examining HIVST among refugee youth. Urbanization of refugees is growing globally [20, 21] and Kampala, Uganda's capital city, hosts >107,000 of Uganda's refugees as of 2022 [22]. Many refugees in Kampala live in informal settlements, including slums [23, 24, 25, 26], and experience living conditions characterized by poverty, overcrowding, violence and disproportionately high HIV prevalence [27, 28, 29, 30, 31, 32, 33, 34]. Qualitative findings with refugee youth in Kampala identified HIV testing barriers, including intersecting stigma [8], inequitable relationship dynamics [35] and other barriers (e.g. low literacy and language barriers) [11]. HIVST may potentially reduce some of these barriers and warrants examination with refugee youth in Kampala.

While HIVST is linked with increased HIV testing uptake, there remain barriers to linkage to HIV care following a positive test that may be mitigated through digital support [36]. Systematic review findings suggest that mobile health (mHealth) approaches (e.g. mobile applications and text messaging [SMS]) can increase HIV testing uptake, including among marginalized communities [37]. Interactive two‐way SMS are particularly relevant for communities with low technical literacy and limited internet/smartphone access, such as refugees in East Africa [38]. SMS reminders increased HIVST uptake in Kenya with truck drivers [39] and sex workers [40], and supported HIVST among persons impacted by hurricanes in Puerto Rico [41]. A population‐based survey in Zimbabwe also reported user preferences for telephone support before using HIVST [42]. Yet, knowledge gaps remain regarding the feasibility of HIVST delivery approaches with refugees, including mHealth.

To address this gap, the Tushirikiane (“supporting each other” in Swahili) trial examined if participating in one of two HIVST delivery approaches (HIVST alone; HIVST combined with mHealth [two‐way supportive SMS]) in comparison with the standard of care (SOC) was associated with increased HIV testing uptake among refugee youth (aged 16–24 years) living in informal settlements in Kampala, Uganda. We assessed changes in primary outcomes (HIV testing uptake; HIV status knowledge; linkage to confirmatory HIV testing; linkage to HIV care; HIV self‐test kit use) and secondary outcomes (depression; condom use self‐efficacy; consistent condom use; sexual relationship power [SRP]; HIV stigma; adolescent sexual and reproductive health [SRH] stigma). We also assessed study pragmatism across PRECIS‐2 dimensions [43] and share lessons learned.

## 2 METHODS

Between 2020 and 2021, we conducted a three‐arm controlled trial across five informal settlements where many refugees in Kampala live [24, 25, 44, 45] grouped into three sites that were randomized in a 1:1:1 method to one of three study arms. Data were collected at three time points (baseline enrolment [T0], 8 months [T1] and 12 months after enrolment [T2]), and participants in intervention arms were also surveyed at a 16‐month follow‐up [T3]. Full details of the trial protocol and decisions on data collection time points are published [46], and the trial is registered with ClinicalTrials.gov (NCT04504097).

### 2.1 Ethical considerations

The trial protocol was approved by the Research Ethics Board from the University of Toronto (Protocol Number: 37496), Mildmay Uganda Research Ethics Committee (Ref: 0806–2019) and Uganda National Council for Science & Technology (Ref: HS2716). All participants provided written informed consent with support from a peer navigator (PN) prior to enrolment.

### 2.2 Participants and recruitment

The informal settlements (Kabalagala, Kansanga, Katwe, Nsambya and Rubaga) were purposively selected because they host many displaced/refugee persons in Kampala [24]. Settlements were grouped into three sites based on proximity (1: Kabalagala and Kansanga; 2: Katwe and Nsambya; 3: Rubaga). Participants were conveniently sampled with approximately equal representation across the three sites with the support of PN, self‐identified refugees with experience working as health/peer educators. The role of PN is further detailed in the study protocol [46], but in brief involved assisting with recruitment, retention, feedback on study and survey design, and implementation support. Refugee youth aged 16–24 years were eligible for inclusion if they lived in one of five target informal settlements, spoke one of the study languages (English, French, Swahili, Luganda, Kinyarwanda or Kirundi) and had access to a mobile phone. Most (61%) refugee youth in this urban context own mobile phones [47].

### 2.3 Intervention and control conditions

Sites were randomized to one of the three arms: (1) HIVST; (2) HIVST and mHealth (bidirectional supportive SMS) (referred to as HIVST+); and (3) SOC (clinic‐based HIV testing), detailed elsewhere [46]. At each time point, the HIVST arm was provided with an HIVST package that included an HIV self‐test kit (OraQuick Rapid HIV‐1/2 Antibody Test, OraSure Technologies), pictorial and written instruction sheet, condoms, lubricant, information booklet and referral cards with local clinic contact information for confirmatory testing. The HIVST+ arm received the same package at each time point, and weekly check‐in messages asking “how are you?” in the participant's study language of choice through a web‐based SMS platform hosted by WelTel [48]. Participants who did not respond or who responded they had a problem were followed‐up by PN. The SOC arm received information about HIV services offered cost‐free at local clinics and Mulago Hospital at each time point.

### 2.4 Assessment and outcomes

**Figure 1:**
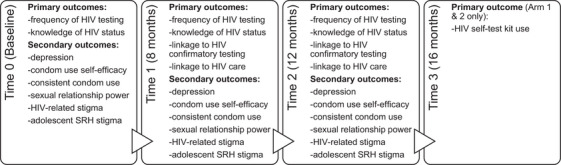
Overview of primary and secondary outcome variables collected across time points in the Tushirikiane Trial, Kampala, Uganda.

Data were collected by in‐person or phone‐based visits using standardized questionnaires administered by trained research assistants. Interviews were conducted in all study languages and data were recorded using a tablet‐based survey application (QuickTapSurvey, Formstack, Toronto, Canada at T0; SurveyCTO, Doblity, Cambridge, USA at T1, T2, T3). Data on demographic, socio‐economic and sexual history were collected at T0. Data on primary outcomes and secondary outcomes were collected at each relevant time point, as detailed in Figure 1.

#### 2.4.1 Primary outcomes: HIV prevention measures

HIV testing uptake was measured as participants’ self‐reported last HIV test. Data were collected at each time point (baseline [T0], 8 months [T1] and 12 months [T2]). At T0, participants were asked if they had an HIV test in the past year; at follow‐up visits, they were asked if they had taken an HIV test (including the use of HIVST) since the previous survey. Participants self‐reported their current HIV status, and at T2, participants were offered a voluntary rapid HIV test. Among those who agreed to take the test (*n* = 314) at T2, HIV status knowledge was defined as correct for those who correctly reported their HIV status prior to receiving the result, and incorrect for those who did not know their status or whose reported status did not match their result. Among the intervention arm participants who reported a positive HIVST result, linkage to confirmatory HIV testing was measured at T1 and T2. Among participants who seroconverted during the study period, linkage to HIV care was measured at T1 and T2 as participants’ self‐reported frequency of accessing HIV care service. Among participants in intervention arms, HIVST kit use was measured at T3 as participants’ self‐reported number of leftover kits and/or if they shared/sold their kits. To reduce social desirability bias, participants were informed there were no wrong answers and that the information they provided would enable the team to better understand community needs for HIVST.

#### 2.4.2 Secondary outcomes: health and wellbeing measures

The patient health questionnaire 9‐item (PHQ‐9) scale was used to measure depression symptoms at each time point [49]. Safer sex efficacy was assessed using the Condom Use Self‐Efficacy Scale (CUSES) [50, 51]. Consistent condom use was assessed using a single question measuring the self‐reported frequency of consistent condom use (always vs. not always). SRP was assessed using the Relationship Control Sub‐Scale from the Sexual Relationship Power (SRP) Scale [52]. HIV stigma (α = 0.83) was assessed using Steward et al.’s 10‐item perceived HIV stigma subscale [53]. Adolescent sexual and reproductive health (SRH) stigma was assessed using the Ugandan version [9] of the Adolescent SRH Stigma scale [54]. The study's scale reliability scores are reported elsewhere [55]. Due to data collection issues leading to differential item non‐response by study arms, scales, including the CUSES (modified scale, six of eight original items): α = 0.90; Adolescent SRH Stigma (modified scale, 12 of 14 original items): α = 0.86 and SRP Scale (modified scale, 14 of 15 original items): α = 0.89, were modified to use only questions asked to all participants.

### 2.5 Power and sample size

Sizes of 144 per study arm (*n* = 432) were required to have 80% power (*p*⩽0.05) to detect a 25% difference (39% vs. 64% tested) in HIV testing between any two groups from three pairwise comparisons (control vs. HIVST; control vs. HIVST+; and HIVST vs. HIVST+), assuming 10% attrition and an intraclass correlation coefficient of 0.013 [56]. These computations were performed using RStudio version 3.3.0 (RStudio Team) and published formulae for multiple comparisons [57].

### 2.6 Statistical analyses

Analyses used intention‐to‐treat principles and methods according to our published protocol [46]. We used descriptive statistics to characterize the study population at baseline in terms of socio‐demographic characteristics. Participant characteristics were compared between SOC and intervention arms using Chi‐squared or Fisher's exact tests for categorical variables and *t*‐tests for continuous variables. We also investigated differences in socio‐demographic and baseline outcome variables between participants retained and those lost to follow‐up (LTFU) using descriptive statistics.

Analyses included all available data from all participants. We conducted difference‐in‐differences models using the full panel data, with the outcome variable regressed on a categorical variable of site, a categorical variable for time point, and an interaction term between these two variables [58, 59]. We used generalized estimating equation models (GEEs) with robust standard errors to estimate intervention effects across time accounting for within‐subject correlation using an unstructured correlation matrix. Using GEE with a working correlation structure and robust standard errors mitigates the bias that may arise from the clustering of repeated observations from the same individual [60]. Logistic GEE regression models were used for categorical outcomes (HIV testing, consistent condom use) and linear GEE regression models were used for continuous outcomes (depression, condom use self‐efficacy, SRP, HIV stigma, adolescent SRH stigma). In these models, the main coefficient on the interaction term reveals the mean difference between each intervention arm and SOC arm, controlling for baseline differences (gender, place of birth, employment status, income security, relationship status) and secular trends [58]. For dichotomous variables that represented a change from baseline, logistic regression models were used to examine the effect of intervention status on outcomes at each time point. Each model was first conducted without adjustment, then with adjustment for gender and age, which were specified *a priori*, as well as characteristics with baseline imbalances.

Analyses focused on the estimate and significance of the interaction term. Difference‐in‐differences intervention effects are expressed as crude and adjusted odds ratios (aORs) or β coefficients (aβ), along with 95% confidence intervals (CIs) [59]. To increase interpretability due to large intervention effects, we also present binary outcomes as predicted probabilities and continuous outcomes as marginal means. All regression analyses were performed as a complete case analysis. For scale outcomes with missing item data, we used participant mean imputation by assigning the mean of the answered items to the missing items. Participant mean imputation has been shown to be valid and produce unbiased results when implemented for missing scale items [61, 62]. All analyses were two‐sided with a significance level of *p* ≤ 0.05 for primary outcomes and using Bonferroni adjustment for secondary outcomes (six outcomes; *p* ≤ 0.008). All analyses were conducted in Stata 16.1 (StataCorp, College Station, TX).

## 3 RESULTS

**Figure 2:**
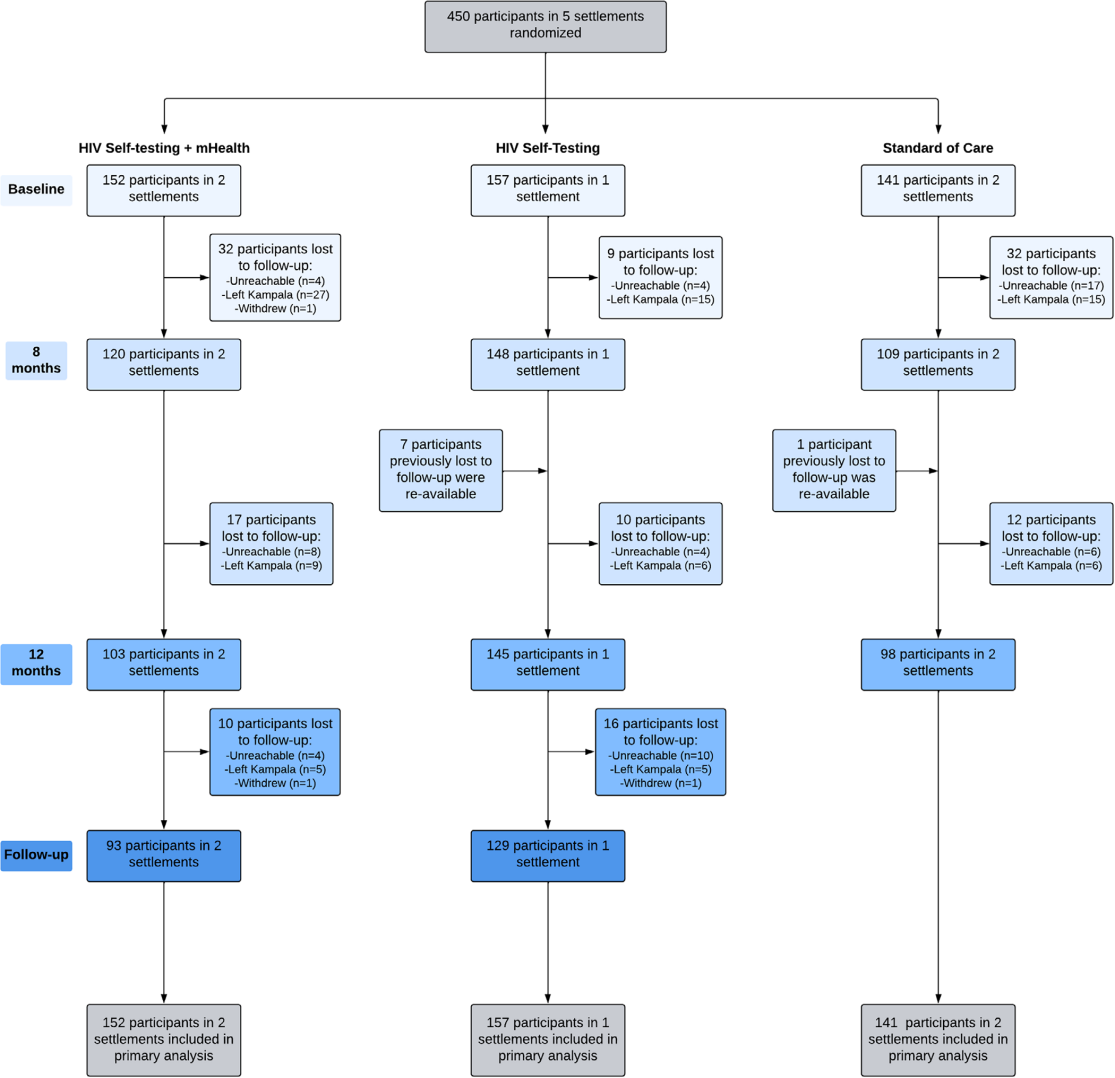
Flow chart of participation of refugee youth enrolled in the Tushirikiane Trial, Kampala, Uganda.

A total of 450 displaced and refugee youth across three sites were enrolled into the trial and assigned to one of three study arms (Figure 2).

Baseline characteristics were similar between study arms, with some exceptions: gender, place of birth, employment status, income security and relationship status (Table 1).

**Table 1:** Baseline characteristics of participants enrolled in the Tushirikiane Trial, Kampala, Uganda, 2020–2021

	Total	SOC	HIVST	HIVST+mHealth
**Intervention arms and participants**				
Settlements	5	2	1	2
Participants	*n* = 450	*n* = 141	*n* = 157	*n* = 152
**Demographic characteristics**				
**Age, mean (SD), y**	20.0 (2.4)	19.8 (2.4)	20.2 (2.5)	20.0 (2.4)
**Gender, *N* (%)**				
Man (cisgender)	228 (50.7)	82 (58.2)	82 (52.2)	64 (42.1)
Woman (cisgender)	219 (48.7)	59 (41.8)	72 (45.9)	88 (57.9)
Transgender woman	3 (0.7)	0 (0.0)	3 (1.9)	0 (0.0)
**Place of birth, *N* (%)** ^a^				
Democratic Republic of Congo	317 (70.4)	106 (75.2)	65 (41.4)	146 (96.1)
Burundi	67 (14.9)	2 (1.4)	62 (39.5)	3 (2.0)
Sudan/South Sudan	20 (4.4)	20 (14.2)	0 (0.0)	0 (0.0)
Others	44 (9.8)	11 (7.8)	30 (19.1)	3 (2.0)
**Length of time in Uganda, *N* (%)**				
<1 year	16 (3.6)	4 (2.8)	4 (2.6)	8 (5.3)
1–5 years	240 (53.3)	66 (46.8)	90 (57.3)	84 (55.3)
6–10 years	133 (29.6)	46 (32.6)	47 (29.9)	40 (26.3)
>10 years	61 (13.6)	25 (17.7)	16 (10.2)	20 (13.2)
**Employment status, *N* (%)** ^a^				
No employment	159 (35.3)	27 (19.2)	88 (56.1)	44 (29.0)
Student	159 (35.3)	63 (44.7)	25 (15.9)	71 (46.7)
Employed (paid/unpaid)	124 (27.6)	44 (31.2)	44 (28.0)	36 (23.7)
**Highest level of education, *N* (%)** ^a^				
Less than secondary	110 (24.4)	36 (25.5)	28 (17.8)	46 (30.3)
Some secondary	181 (40.2)	55 (39.0)	65 (41.4)	61 (40.1)
Secondary +	154 (34.2)	48 (34.0)	61 (38.9)	45 (29.6)
**Income secure, *N* (%)** ^a^				
Never (Least income secure)	210 (46.7)	49 (34.8)	126 (80.3)	35 (23.0)
Sometimes	175 (38.9)	75 (53.2)	24 (15.3)	76 (50.0)
Most days	36 (8.0)	8 (5.7)	0 (0.0)	28 (18.4)
Everyday (Most income secure)	27 (6.0)	9 (6.4)	7 (4.5)	11 (7.2)
**Relationship status, *N* (%)** ^a^				
No current partner	189 (42.0)	54 (38.3)	82 (52.2)	53 (34.9)
Dating one partner/married	184 (40.9)	56 (39.7)	55 (35.0)	73 (48.0)
Casual dating/multiple partners	73 (16.2)	28 (19.9)	20 (12.7)	25 (16.5)
**Has dependents, *N* (%)**				
No	406 (90.2)	134 (95.0)	139 (88.5)	133 (87.5)
Yes	44 (9.8)	7 (5.0)	18 (11.5)	19 (12.5)

*Note*: SOC, standard of care; HIVST, HIV self‐test; SD, standard deviation.

^a^ Missing values for place of birth *n* = 2 (0.4%); employment *n* = 8 (1.8%); education *n* = 5 (1.1%); income *n* = 2 (0.4%); relationship *n* = 4 (0.9%).

About half of the participants identified as women (48.7%); participants’ mean (SD) age was 20.0 (2.4) years; and most (70.4%) were from the Democratic Republic of the Congo (DRC). Participant retention dropped between T0 and T1 (83.8%) and T2 (76.9%), with participants reporting leaving the study sites and/or becoming unavailable. A greater proportion of participants LTFU were from the DRC and reported lower income security, potentially exacerbated by COVID‐19 (Tables S1 and S2).

### 3.1 Primary outcomes

At baseline, 31.2% of participants reported a past‐year HIV test, and this proportion was similar within each study arm. HIV testing uptake was substantially higher in both intervention arms compared to SOC at both T1 and T2 (Table 2), including in gender‐stratified analysis (Table S3).

**Table 2:** Distribution of HIV prevention and health and wellbeing outcomes by group and across time points among Tushirikiane Trial participants, Kampala, Uganda, 2020–2021

	Total		SOC		HIVST		HIVST+mHealth	
	*N*	*N* (%) or mean (standard deviation [SD]) (%)	*N*	*N* (%) or mean (SD)	*N*	*N* (%) or mean (SD)	*N*	*N* (%) or mean (SD)
***Primary outcomes***								
** HIV testing, *n* (%)**								
Baseline [T0]	449	140 (31.2)	141	50 (35.5)	156	43 (27.6)	152	47 (30.9)
8 months [T1]	377	275 (72.9)	109	27 (24.8)	148	135 (91.2)	120	113 (94.2)
12 months [T2]	346	276 (79.8)	98	36 (36.7)	145	143 (98.6)	103	97 (94.2)
** HIV status knowledge, *n* (%)**								
Baseline [T0]	–	–	–	–	–	–	–	–
8 months [T1]	–	–	–	–	–	–	–	–
12 months [T2]	314	275 (87.6)	96	59 (61.5)	121	121 (100.0)	97	95 (97.9)
** Linkage to HIV confirmatory testing** ^a^ **, *n* (%)**								
Baseline [T0]	–	–	–	–	–	–	–	–
8 months [T1]	1	0 (0.0)	–	–	0	0 (0.0)	1	0 (0.0)
12 months [T2]	0	0 (0.0)	–	–	0	0 (0.0)	0	0 (0.0)
** Linkage to HIV care** ^b^ **, *n* (%)**								
Baseline [T0]	–	–	–	–	–	–	–	–
8 months [T1]	1	0 (0.0)	0	0 (0.0)	0	0 (0.0)	1	0 (0.0)
12 months [T2]	0	0 (0.0)	0	0 (0.0)	0	0 (0.0)	0	0 (0.0)
** Unused HIVST kits, *n* (%)**								
Baseline [T0]	–	–	–	–	–	–	–	–
8 months [T1]	–	–	–	–	–	–	–	–
12 months [T2]	–	–	–	–	–	–	–	–
Follow‐up at 16 months [T3]	222	43 (19.4)	–	–	129	3 (2.3)	93	40 (43.0)
***Secondary outcomes***								
** Depression PHQ‐9, mean (SD)**								
Baseline [T0]	449	5.8 (5.8)	141	3.5 (5.2)	157	9.2 (6.0)	151	4.4 (4.4)
8 months [T1]	372	6.2 (6.2)	105	5.1 (4.4)	147	7.5 (7.3)	120	5.6 (5.7)
12 months [T2]	346	5.9 (5.8)	98	4.6 (4.7)	145	7.0 (7.0)	103	5.7 (4.5)
** Sexual relationship power, mean (SD)**								
Baseline [T0]	450	42.1 (9.1)	141	42.8 (9.7)	157	38.8 (8.1)	152	44.8 (8.5)
8 months [T1]	372	43.4 (9.5)	109	43.6 (9.0)	146	42.8 (10.3)	117	44.0 (8.8)
12 months [T2]	342	45.5 (10.3)	97	47.0 (9.5)	145	44.1 (12.7)	100	46.0 (5.9)
** Condom use self‐efficacy, mean (SD)**								
Baseline [T0]	450	14.3 (5.1)	141	12.4 (5.6)	157	15.9 (3.8)	152	14.4 (5.3)
8 months [T1]	373	16.4 (5.8)	109	15.6 (4.5)	146	16.8 (6.6)	118	16.6 (5.7)
12 months [T2]	345	15.3 (6.0)	98	14.9 (5.4)	145	14.8 (7.0)	102	16.5 (4.6)
** Consistent condom use** ^c^, ^d^ **, *n* (%)**								
Baseline [T0]	–	–	–	–	–	–	–	–
8 months [T1]	104	29 (27.9)	23	8 (34.8)	41	11 (26.8)	40	10 (25.0)
12 months [T2]	113	24 (21.2)	35	10 (28.6)	53	7 (13.2)	25	7 (28.0)
** HIV‐related stigma, mean (SD)**								
Baseline [T0]	450	21.2 (5.6)	141	21.6 (5.7)	157	20.4 (6.1)	152	21.6 (4.8)
8 months [T1]	377	22.5 (5.4)	109	20.9 (5.3)	148	24.3 (5.1)	120	21.7 (5.1)
12 months [T2]	346	23.6 (5.5)	98	23.3 (5.8)	145	24.9 (5.7)	103	22.1 (4.3)
** Adolescent SRH stigma, mean (SD)**								
Baseline [T0]	450	9.2 (3.2)	141	8.2 (2.8)	157	8.8 (4.0)	152	10.5 (1.9)
8 months [T1]	377	10.0 (2.4)	109	8.2 (2.3)	148	11.3 (1.5)	120	10.1 (2.3)
12 months [T2]	346	9.2 (2.9)	98	8.6 (2.6)	145	10.6 (2.3)	103	7.9 (3.1)
**mHealth usage**								
				**Used WelTel, *N* (%)**				
						No		41 (39.81)
						Yes		62 (60.19)
				**Reason for not using WelTel, *N* = 39, (%)**				
					Phone not working			2 (5.13)
					No mobile data			3 (7.69)
					Used a different phone number			30 (76.92)
					Messages not received			4 (10.26)

Abbreviations: HIVST, HIV self‐test; PHQ‐9, patient health questionnaire; SOC, standard of care; SRH, sexual and reproductive health.

^a^ Among those reporting a positive HIV self‐test.

^b^ Among those who serconverted during the study.

^c^ Among those reporting being sexually active in the past 3 months.

^d^ Data error at baseline, so data were only collected at 8 and 12 months.

**Figure 3:**
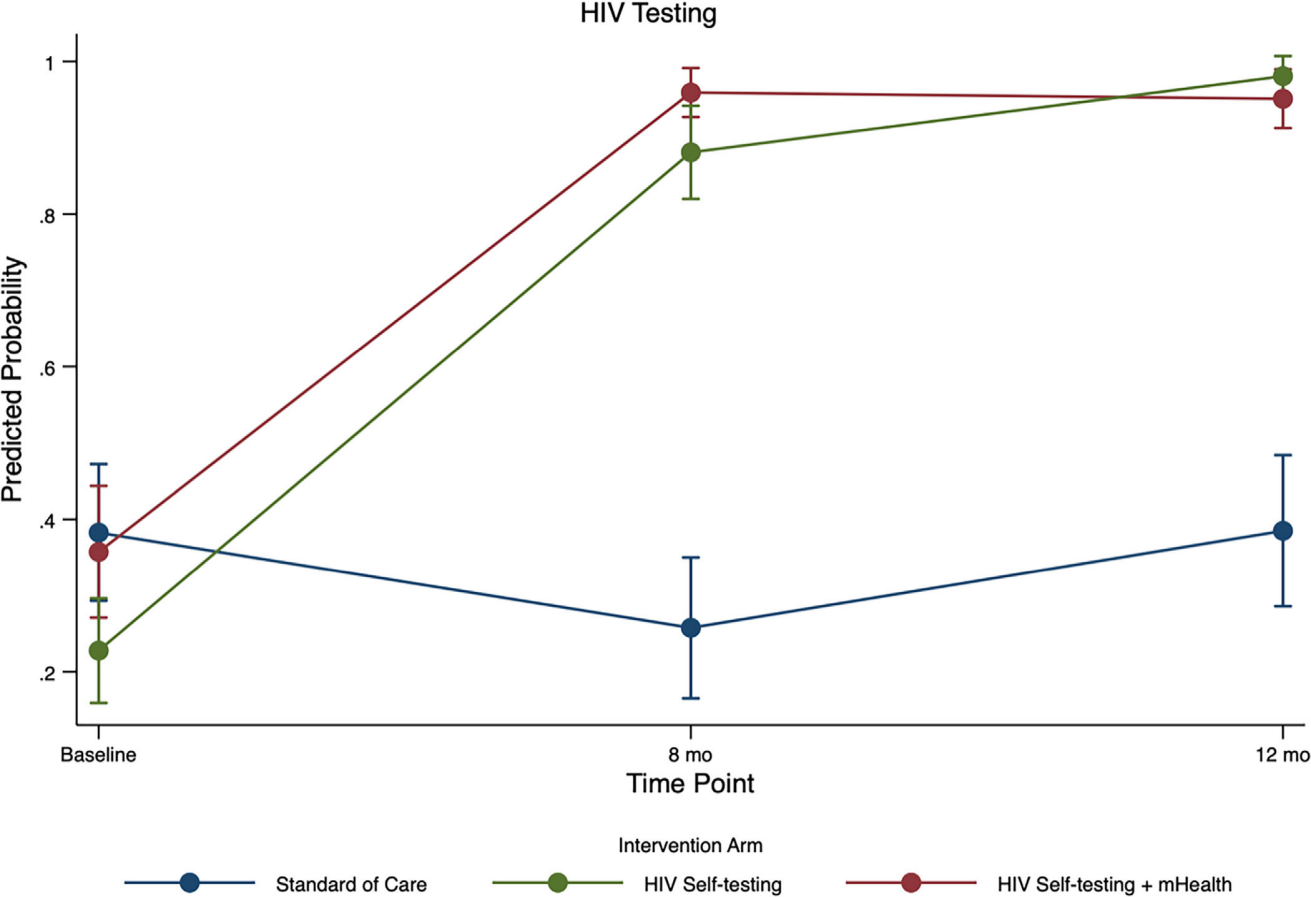
Estimated predicted probabilities and confidence intervals (CI) for time by treatment group interaction effects for primary HIV testing outcome among Tushirikiane Trial participants, Kampala, Uganda, 2020–2021. The blue line is standard of care, the green line is HIV self‐test and the red line is HIV self‐test + mHealth arm.

In difference‐in‐difference regression models using HIV testing data at all time points, the HIVST arm had significantly higher odds of testing at T1 (27.6% [*n* = 43] at T0 vs. 91.2% [*n* = 135] at T1; aOR 64.5; 95% CI: 26.3,159.0) as did the HIVST+ arm (30.9% [*n* = 47] at T0 vs. 94.2% [*n* = 113] at T1; aOR 106.8; 95% CI: 36.0, 316.6) than the SOC arm (35.5% [*n* = 50] at T0 vs. 24.8% [*n* = 27] at T1) (Table 3). This remained high at T2, with predicted probabilities in intervention arms approaching 100% (Figure 3). In the HIVST+ arm, 60.2% (*n* = 62) of participants reported having responded to the two‐way messages throughout the intervention. Among those who did not respond, the most reason was having changed phone numbers during the study (*n* = 30; 76.92%) (Table 2).

**Table 3:** Difference‐in‐difference estimates for effectiveness of HIV self‐testing and mHealth interventions versus standard of care on primary HIV prevention outcomes among Tushirikiane Trial participants, Kampala, Uganda, 2020–2021

	OR	95% CI	*p*‐Value	aOR^*^	95% CI	*p*‐Value
**Uptake of HIV testing** ^a^						
** Intervention effects at 8 months [T1]**						
HIVST versus SOC	45.0	19.7, 102.6	<0.001	64.5	26.3, 159.0	<0.001
HIVST+mHealth versus SOC	61.1	23.2, 160.7	<0.001	106.8	36.0, 316.6	<0.001
**Intervention effects at 12 months [T2]**						
HIVST versus SOC	176.4	38.1, 816.5	<0.001	245.5	50.1, 1204.1	<0.001
HIVST+mHealth versus SOC	34.1	12.4, 93.8	<0.001	45.9	15.5, 135.7	<0.001
**HIV status knowledge** ^b^						
**Intervention effects at 12 months** ^**^ **[T2]**						
SOC	ref			ref		
HIVST and HIVST + mHealth	67.7	15.9, 289.2	<0.001	101.2	18.3, 561.0	<0.001
**Unused HIVST kits** ^c^						
**Intervention effects at 16 months follow‐up [T3]**						
HIVST	ref			ref		
HIVST + mHealth	31.7	9.4, 107.0	<0.001	22.7	4.4, 118.2	<0.001

Abbreviations: CI, confidence interval; HIVST, HIV self‐test; OR, odds ratio; SOC, standard of care.

^a^ Intervention effect on uptake of HIV testing is estimated as the interaction between intervention arm and time point, calculated using generalized estimating equation logistic regression models with an unstructured correlation matrix.

^b^ Intervention effect on HIV status knowledge is only measured at 12 months for both intervention arms compared to SOC, calculated using logistic regression models.

^c^ Intervention effect on use of HIVST kits is measured at 16 months follow‐up for HIVST+mHealth arm compared to HIVST arm, calculated using logistic regression models.

* Adjusted for pre‐specified covariates (age, gender) and baseline imbalances (birth country, employment, income security, relationship status).

** HIVST and HIVST+mHealth were combined in regression analyses for HIV status knowledge given 100% status knowledge among the HIVST arm.

Similarly, HIV status knowledge verified by point‐of‐care testing (measured at T2) was substantially higher in both intervention arms (HIVST: 100% [*n* = 121], HIVST+: 97.9% [*n* = 95]) compared with SOC (61.5% [*n* = 59]) (Table 2). Given that 100% of youth in the HIVST arm correctly measured HIV status knowledge, results from both intervention arms were combined and compared to SOC in regression analyses. Results indicate a significantly greater odds of correctly reporting HIV status knowledge among intervention arms than those in SOC (aOR 101.2; 95% CI: 18.3 561.0) (Table 3). One participant reported seroconverting as determined by HIVST during the study period (Table 2); there were no additional HIV cases identified in point‐of‐care testing. We were unable to examine the intervention effects’ linkage to HIV confirmatory testing or to HIV care.

At T3, intervention arm participants were asked if they had any unused HIVST from the study. The HIVST+ arm reported significantly higher odds of unused tests than those in the HIVST arm (aOR 22.7; 95% CI: 4.4, 118.2) (Tables 2 and 3).

### 3.2 Secondary outcomes

There were modest changes in secondary outcomes across time in intervention arms (Table 2 and Figure S1). In regression models using data at all time points, the HIVST arm had significantly reduced depression scores at T1 than the SOC, and this persisted at T2 (Table 4). There were increases in SRP among all arms, with only a significant difference between the HIVST and SOC arms at T1 (Tables 2 and 4). There was a significant decrease in condom use self‐efficacy in the HIVST arm compared to the SOC, but no significant changes in consistent condom use (Table 4). The HIVST arm reported increased HIV‐related stigma and adolescent SRH stigma at T1 and T2; however, the HIVST+ arm reported a significant decrease in adolescent SRH stigma scores at T2 (Table 4).

**Table 4:** Difference‐in‐difference estimates for effectiveness of HIV self‐testing and mHealth interventions versus standard of care on secondary health and wellbeing outcomes among Tushirikiane Trial participants, Kampala, Uganda, 2020–2021

	aβ^a^	95% CI	*p*‐Value^b^
**Depression PHQ‐9**			
**Intervention effects at 8 months [T1]**			
HIVST versus SOC	−3.21	−4.71, −1.71	**<0.001**
HIVST+mHealth versus SOC	−0.38	−1.93, 1.17	0.630
**Intervention effects at 12 months [T2]**			
HIVST versus SOC	−3.24	−4.81, −1.67	**<0.001**
HIVST+mHealth versus SOC	−0.04	−1.65, 1.57	0.963
**Sexual relationship power**			
**Intervention effects at 8 months [T1]**			
HIVST versus SOC	3.39	0.56, 6.23	**0.019**
HIVST+mHealth versus SOC	−0.95	−3.69, 1.80	0.499
**Intervention effects at 12 months [T2]**			
HIVST versus SOC	0.98	−2.14, 4.11	0.538
HIVST+mHealth versus SOC	−2.52	−5.15, 0.11	0.061
**Condom use self‐efficacy**			
**Intervention effects at 8 months [T1]**			
HIVST versus SOC	−2.26	−3.79, −0.73	**0.004**
HIVST+mHealth versus SOC	−0.92	−2.45, 0.62	0.243
**Intervention effects at 12 months [T2]**			
HIVST versus SOC	−3.78	−5.55, −2.00	**<0.001**
HIVST+mHealth versus SOC	−0.62	−2.18, 0.95	0.440
**HIV‐related stigma**			
**Intervention effects at 8 months [T1]**			
HIVST versus SOC	4.78	3.14 6.42	**<0.001**
HIVST+mHealth versus SOC	1.06	−0.55, 2.67	0.197
**Intervention effects at 12 months [T2]**			
HIVST versus SOC	2.95	1.09, 4.81	**0.002**
HIVST+mHealth versus SOC	−1.13	−2.79, 0.53	0.182
**Adolescent SRH stigma**			
**Intervention effects at 8 months [T1]**			
HIVST versus SOC	2.58	1.67, 3.49	**<0.001**
HIVST+mHealth versus SOC	−0.38	−1.15, 0.39	0.328
**Intervention effects at 12 months [T2]**			
HIVST versus SOC	1.40	0.43, 2.36	**0.005**
HIVST+mHealth versus SOC	−3.04	−4.01, −2.08	**<0.001**

	**aOR** ^a^	**95% CI**	***p*‐Value** ^b^
**Consistent condom use**			
**Intervention effects at 8 months [T1]**			
HIVST versus SOC	–	–	–
HIVST+mHealth versus SOC	–	–	–
**Intervention effects at 12 months [T2]**			
HIVST versus SOC	0.95	0.23, 3.89	0.946
HIVST+mHealth versus SOC	1.71	0.30, 9.61	0.543

Abbreviations: CI, confidence interval; HIVST, HIV self‐testing; OR, odds ratio; PHQ‐9, patient health questionnaire; SOC, standard of care; SRH, sexual and reproductive health.

^a^ Intervention effect is estimated as the interaction between intervention arm and time point, calculated using generalized estimating equation linear or logistic regression models with an unstructured correlation matrix. Estimates adjusted for pre‐specified covariates (age, gender) and baseline imbalances (birth country, employment, income security, relationship status).

^b^ Statistical significance set at *p*⩽ 0.008 due to Bonferroni adjustment.

We retrospectively assessed the degree of pragmatism following study completion [63], whereby three investigators completed the PRECIS‐2 tool [43]; findings (Figure S2) reveal that the Tushirikiane trial employed both pragmatic (eligibility criteria, setting, organization, outcome, analysis) and explanatory approaches (recruitment path, flexibility of delivery flexibility, adherence flexibility, follow‐up) (Table S4).

## 4 DISCUSSION

Findings reveal the potential of HIVST and mHealth to address refugee youth's HIV testing needs in an urban Ugandan context. Among this sample of urban refugee youth, two‐thirds of whom had not tested in the year before the intervention, testing uptake and status knowledge in the HIVST and HIVST+ arms were higher (<90%) compared to those receiving SOC at 8 and 12 months (approximately 30%). HIVST offers great promise to contribute to efforts to achieve the UNAIDS goal of 95% of people knowing their status in urban youth refugee communities in Uganda and warrants further exploration in larger trials.

Findings align with prior research on the feasibility and acceptability of HIVST in Uganda among non‐refugees [64, 65, 66], and among non‐refugee youth in other African contexts [17]. Our findings align with evidence in systematic reviews [5, 6, 7], highlighting the critical role of HIVST in increasing access to, and uptake of, HIV testing for marginalized populations. It is plausible that HIVST may address refugee youth HIV testing barriers [10]. As Uganda develops and adopts HIVST as a testing approach for vulnerable youth, our findings suggest that refugee youth are capable of correctly using HIVST to know their HIV status.

There were some modest changes in secondary outcomes, including stigma and depression. Stigma regarding HIV and SRH increased among those receiving HIVST alone, while adolescent SRH stigma decreased among those receiving HIVST alongside mHealth peer support. These findings suggest that youth may encounter stigma when engaging with HIVST, particularly when self‐testing without mHealth support. Prior research suggests that facility‐based HIV testing may increase stigma exposure, potentially through encounters with healthcare workers, family members, community members and/or friends [8], whereas HIVST is usually reported as reducing stigma exposure [6]. While HIVST alone was associated with increases in both HIV‐related and adolescent SRH stigma, these increases were *not* found among participants receiving HIVST alongside mHealth support. Indeed, when offered SMS alongside HIVST, participants reported *reduced* adolescent‐SRH stigma. Plausibly peer support available via SMS helped participants navigate concerns/experiences of stigma and isolation. Hence, peer support may be important alongside HIVST for reducing stigma among urban refugee youth who may experience disrupted social networks [67] and intersecting stigma [8]. Studies have similarly documented preferences for peer support with HIVST in Uganda, including among gay, bMSM [68, 69] and sex workers [70]. Our findings can inform stigma‐informed approaches to HIVST implementation and trial design.

We found that participants in the HIVST arm reported reduced depression, but no change was reported among the HIVST+ group. This could be partially due to only 60% of the mHealth arm engaging with WelTel due to variable technical/economic issues. Future research could explore COVID‐19 impacts on technology access and strategies to enhance health equity in digital clinical trials with urban refugees [71]. Also, prior US‐based research did not identify changes in distress following HIVST [72], and HIV testing was not associated with changes in depression among outpatients in Uganda who tested HIV negative [73]. It is plausible that the peer support offered focused on HIV and the nature/quality of support was not sufficient to address depression. Importantly, there was no difference in consistent condom use by the study arm, suggesting no increased HIVST‐related HIV risk. This corroborates research with sex workers in Uganda that reported no significant difference in condom uptake between HIVST and SOC [74].

There were study limitations that can inform future humanitarian research. First, there was a larger LTFU than anticipated, in part due to the COVID‐19 pandemic. This is a generally mobile population, with high levels of income insecurity exacerbated by the pandemic [75, 76]. Future studies could plan for larger than 10% attrition with urban refugees. Second, there were baseline differences between the study sites. While we adjusted for these baseline differences in analyses, these differences suggest that the socio‐cultural context of study arms—informal settlements in close proximity—should be taken into consideration in interpreting these findings. This also points to the complexity of conducting urban refugee research in a context such as Kampala that hosts refugees from many countries who often live in informal settlements with refugees from similar communities; the nature of this migration results in socio‐cultural differences between informal settlements that we documented. It also raises questions of how (or whether) to reduce heterogeneity in study design by only including refugees from the same country (e.g. DRC), which would reduce pragmatism, or randomizing by individual rather than site, which would increase contamination risks across intervention arms due to closely knit networks of refugees living in slum communities [77]. Additionally, while our analytic approach accounts for clustering of repeated observations within individuals, we are not able to account for clustering between individuals within a site or settlement due to the small number of sites. This social organization and socio‐cultural diversity of urban refugees in Kampala's informal settlements presents challenges in designing a cluster randomized trial that requires multiple clusters (e.g. informal settlements) per condition with minimal baseline differences. Third, the low rate of seropositivity signals that refugee youth reached in this study may not be at high risk of HIV, hence future HIVST studies with urban refugee youth can screen for HIV risk (e.g. transactional sex) and conduct targeted recruitment. This, however, would reduce study design pragmatism. Finally, data on participants screened were not collected, therefore, we were not able to describe the percent meeting eligibility criteria across each site to assess potential biases in the generalizability of these findings. Despite these limitations, this study is unique in its longitudinal design and offers new insight into the feasibility and acceptability of HIVST among urban refugee youth. Future HIVST research with sexually active urban refugee youth could consider pre‐exposure prophylaxis (PrEP) linkage, also understudied among refugee youth [2, 3]. Due to our study limitations (modest sample size, baseline imbalances across informal settlements), there is a need for future larger randomized trials to advance both knowledge of, and methods to assess, HIVST delivery approaches in urban humanitarian settings.

## 5 CONCLUSIONS

This study documented the feasibility, acceptability and promise of HIVST among urban refugee youth with previously undiagnosed HIV. The social organization of diverse urban refugee communities in Kampala presents significant methodological challenges for randomized controlled trials and warrants further exploration and innovation in trial design. These findings can inform future HIVST research in humanitarian settings, including a focus on HIVST complemented with mHealth peer support for marginalized youth.

## COMPETING INTERESTS

RL is an academic physician‐researcher and also has interests in a non‐profit and private company social enterprise, WelTel Inc., that develops and provides digital health software. He is not being paid or otherwise compensated by WelTel for this project. No other authors declare a conflict of interest.

## AUTHORS’ CONTRIBUTIONS

Study design: CHL, MO, DKM, RH, SM, RL, PK, SDB, SN and LM. Data collection: DKM, RH, SM, SB and AN. Data management: CHL, MO, DKM, RH, IB, RL, AN and ML. Manuscript writing: CHL, MO, IB, ML, SDB and RL. Manuscript editing: CHL, MO, IB, RH, DKM, AN, SM, PK, ML, SB, SDB, RL, SN, KN and LM.

## FUNDING

This study has been funded by the Canadian Institutes of Health Research (project grant 389142). Funding agencies played no role in the design or execution of the study. CL is also funded by the Canada Research Chairs program (Tier 2: Logie), Canada Foundation for Innovation (Logie's SSHINE Lab), and the Ontario Ministry of Research and Innovation (ERA: Logie).

## Supporting information

Supporting Information

**Figure S2** PRECIS‐2 wheel illustrating the retrospective assessment of Tushirikiane study pragmatism

Supporting Information

## Data Availability

The data that support the findings of this study are available on request from the corresponding author. The data are not publicly available due to privacy and ethical restrictions.
