# Supplementary material for: Findings from the Tushirikiane mobile health (mHealth) HIV self‐testing pragmatic trial with refugee adolescents and youth living in informal settlements in Kampala, Uganda
Source: J Int AIDS Soc. 2023 Oct 18;26(10):e26185. doi: 10.1002/jia2.26185 (PMC10583643; doi:10.1002/jia2.26185)
Supplement: Supplementary file 1 — Supporting Information [file JIA2-26-e26185-s001.docx]

**Supplemental Figure 1.** Estimated marginal means or predicted probabilities and confidence intervals (CI) for time by treatment group interaction effects for secondary health and well-being outcomes among Tushirikiane Trial participants, Kampala, Uganda, 2020-2021. Blue line is standard of care, green line is HIV self-test, and red line is HIV self-test + mHealth arm.
