# Supplementary figures and images for: Findings from the Tushirikiane mobile health (mHealth) HIV self‐testing pragmatic trial with refugee adolescents and youth living in informal settlements in Kampala, Uganda
Source: J Int AIDS Soc. 2023 Oct 18;26(10):e26185. doi: 10.1002/jia2.26185 (PMC10583643; doi:10.1002/jia2.26185)

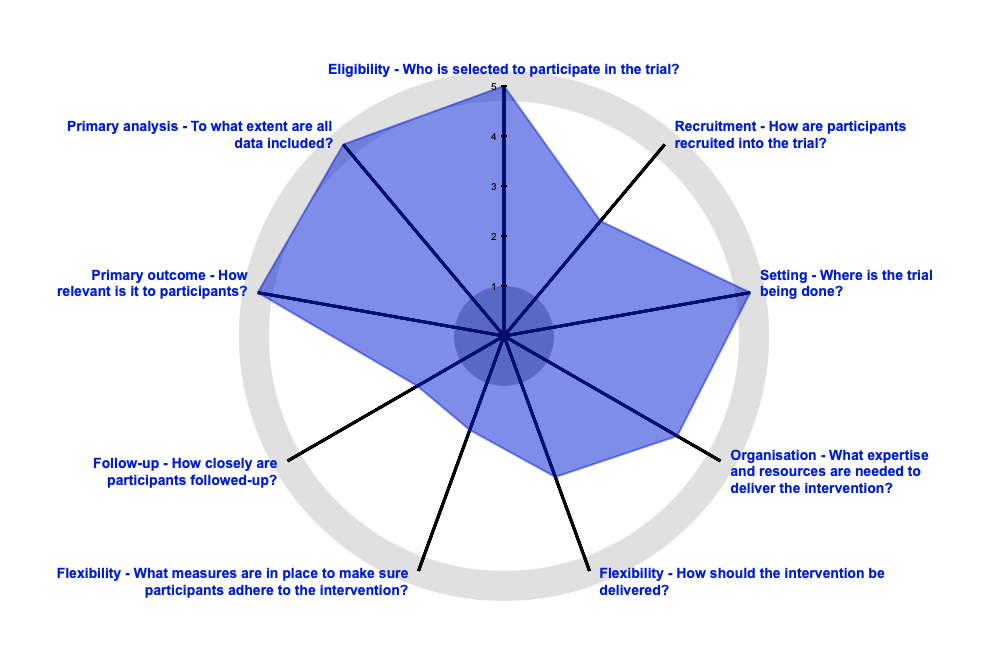

Supplement: Supplementary file 2 — Figure S2 PRECIS‐2 wheel illustrating the retrospective assessment of Tushirikiane study pragmatism [file JIA2-26-e26185-s003.png]
