# Supplementary material for: Findings from the Tushirikiane mobile health (mHealth) HIV self‐testing pragmatic trial with refugee adolescents and youth living in informal settlements in Kampala, Uganda
Source: J Int AIDS Soc. 2023 Oct 18;26(10):e26185. doi: 10.1002/jia2.26185 (PMC10583643; doi:10.1002/jia2.26185)
Supplement: Supplementary file 3 — Supporting Information [file JIA2-26-e26185-s002.docx]

**Supplemental Table 1**. Tushirikiane Trial participant loss to follow-up at 8 months

|  |  |  | **Retained** | **LTFU at 8 mo** | **p-value** |
| --- | --- | --- | --- | --- | --- |
|  |  |  | n=377 | n=73 |  |
| **Demographic Characteristics** | | |  |  |  |
|  | **Age, mean (SD), y** | | 20.1 (2.4) | 19.8 (2.5) | 0.291 |
|  | **Gender, N (%)** | |  |  | 0.468 |
|  |  | Man (cisgender) | 187 (49.6) | 41 (56.2) |  |
|  |  | Woman (cisgender) | 187 (49.6) | 32 (43.8) |  |
|  |  | Transgender | 3 (0.8) | 0 (0.0) |  |
|  | **Place of Birth, N (%)** | |  |  | 0.003 |
|  |  | Democratic Republic of Congo | 254 (67.4) | 63 (88.7) |  |
|  |  | Burundi | 62 (16.7) | 4 (5.6) |  |
|  |  | Sudan / South Sudan | 19 (5.0) | 1 (1.4) |  |
|  |  | Others | 41 (10.9) | 3 (4.2) |  |
|  | **Length of time in Uganda, N (%)** | |  |  | 0.774 |
|  |  | <1 year | 12 (3.2) | 4 (5.5) |  |
|  |  | 1-5 years | 202 (53.6) | 38 (52.1) |  |
|  |  | 6-10 years | 112 (29.7) | 21 (28.8) |  |
|  |  | >10 years | 51 (13.5) | 10 (13.7) |  |
|  | **Employment Status, N (%)** | |  |  | 0.963 |
|  |  | No employment | 133 (35.6) | 26 (36.1) |  |
|  |  | Student | 134 (36.2) | 25 (34.7) |  |
|  |  | Employed (paid/unpaid) | 103 (27.8) | 21 (29.2) |  |
|  | **Highest Level of Education, N (%)** | |  |  | 0.172 |
|  |  | Less than secondary | 86 (23.1) | 24 (33.3) |  |
|  |  | Some secondary | 154 (41.3) | 27 (37.5) |  |
|  |  | Secondary + | 133 (35.7) | 21 (29.2) |  |
|  | **Income Secure, N (%)** | |  |  | <0.001 |
|  |  | Never (Least income secure) | 189 (50.4) | 21 (28.8) |  |
|  |  | Sometimes | 143 (38.1) | 32 (43.8) |  |
|  |  | Most days | 24 (6.4) | 12 (16.4) |  |
|  |  | Everyday (Most income secure) | 19 (5.1) | 8 (11.0) |  |
|  | **Relationship Status, N (%)** | |  |  | 0.531 |
|  |  | No current partner | 161 (43.2) | 28 (38.4) |  |
|  |  | Dating one partner/married | 154 (41.3) | 30 (41.1) |  |
|  |  | Casual dating/multiple partners | 58 (15.6) | 15 (20.6) |  |
|  | **Children, N (%)** | |  |  | 0.218 |
|  |  | No | 343 (91.0) | 63 (86.3) |  |
|  |  | Yes | 34 (9.0) | 10 (13.7) |  |

**Supplemental Table 2.** Tushirikiane Trial participant loss to follow-up at 12 months

|  |  |  | **Retained** | **LTFU at 12 mo** | **p-value** |
| --- | --- | --- | --- | --- | --- |
|  |  |  | n= 346 | n= 104 |  |
| **Demographic Characteristics** | | |  |  |  |
|  | **Age, mean (SD), y** | | 20.0 (2.5) | 20.0 (2.4) | 0.783 |
|  | **Gender, N (%)** | |  |  | 0.554 |
|  |  | Man (cisgender) | 171 (49.4) | 57 (54.8) |  |
|  |  | Woman (cisgender) | 173 (50.0) | 46 (44.2) |  |
|  |  | Transgender | 2 (0.6) | 1 (1.0) |  |
|  | **Place of Birth, N (%)** | |  |  | <0.001 |
|  |  | Democratic Republic of Congo | 228 (65.9) | 89 (87.3) |  |
|  |  | Burundi | 61 (17.6) | 6 (5.9) |  |
|  |  | Sudan / South Sudan | 16 (4.6) | 4 (3.9) |  |
|  |  | Others | 41 (11.9) | 3 (2.9) |  |
|  | **Length of time in Uganda, N (%)** | |  |  | 0.661 |
|  |  | <1 year | 11 (3.2) | 5 (4.8) |  |
|  |  | 1-5 years | 181 (52.3) | 59 (56.7) |  |
|  |  | 6-10 years | 106 (30.6) | 27 (26.0) |  |
|  |  | >10 years | 48 (13.9) | 13 (12.5) |  |
|  | **Employment Status, N (%)** | |  |  | 0.486 |
|  |  | No employment | 127 (37.4) | 32 (31.4) |  |
|  |  | Student | 118 (34.7) | 41 (40.2) |  |
|  |  | Employed (paid/unpaid) | 95 (27.9) | 29 (28.4) |  |
|  | **Highest Level of Education, N (%)** | |  |  | 0.200 |
|  |  | Less than secondary | 79 (23.1) | 31 (30.1) |  |
|  |  | Some secondary | 138 (40.4) | 43 (41.8) |  |
|  |  | Secondary + | 125 (36.6) | 29 (28.2) |  |
|  | **Income Secure, N (%)** | |  |  | <0.001 |
|  |  | Never (Least income secure) | 177 (51.5) | 33 (31.7) |  |
|  |  | Sometimes | 131 (38.1) | 44 (42.3) |  |
|  |  | Most days | 17 (4.9) | 19 (18.3) |  |
|  |  | Everyday (Most income secure) | 19 (5.5) | 8 (7.7) |  |
|  | **Relationship Status, N (%)** | |  |  | 0.265 |
|  |  | No current partner | 150 (43.9) | 39 (37.5) |  |
|  |  | Dating one partner/married | 141 (41.2) | 43 (41.4) |  |
|  |  | Casual dating/multiple partners | 51 (14.9) | 22 (21.2) |  |
|  | **Children, N (%)** | |  |  | 0.286 |
|  |  | No | 315 (91.0) | 91 (87.5) |  |
|  |  | Yes | 31 (9.0) | 13 (12.5) |  |

**Supplemental Table 3.** Gender-stratified effectiveness of HIV self-testing and mHealth interventions on primary HIV testing outcomes among Tushirikiane Trial participants, Kampala, Uganda, 2020-2021

|  |  |  | **OR** | **95% CI** | **p-value** | **aOR*** | **95% CI** | **p-value** |
| --- | --- | --- | --- | --- | --- | --- | --- | --- |
| **Cisgender men** | | |  |  |  |  |  |  |
| **Uptake of HIV Testing^a^** | | |  |  |  |  |  |  |
|  | **Intervention effects at 8 months** | | |  |  |  |  |  |
|  |  | HIVST vs SOC | 20.92 | 6.86, 63.75 | <0.001 | 31.84 | 9.71, 104.41 | <0.001 |
|  |  | HIVST+mHealth vs SOC | 129.97 | 14.94, 1130.3 | <0.001 | 189.75 | 19.34, 1861.28 | <0.001 |
|  | **Intervention effects at 12 months** | | |  |  |  |  |  |
|  |  | HIVST vs SOC | 164.08 | 18.99, 1417.9 | <0.001 | 205.71 | 25.75, 1643.64 | <0.001 |
|  |  | HIVST+mHealth vs SOC | 32.04 | 7.24, 141.76 | <0.001 | 35.22 | 7.65, 162.13 | <0.001 |
| **HIV Status Knowledge^b^** | | |  |  |  |  |  |  |
|  | **Intervention effects at 12 months** | | |  |  |  |  |  |
|  |  | SOC | ref | | | ref | | |
|  |  | HIVST & HIVST + mHealth | 67.33 | 8.67, 522.67 | <0.001 | 66.93 | 6.85, 653.92 | <0.001 |
| **Unused HIVST Kits^c^** | | |  |  |  |  |  |  |
|  | **Intervention effects at 16 months follow-up** | | |  |  |  |  |  |
|  |  | HIVST | ref | | | ref | | |
|  |  | HIVST + mHealth | n/a | | | n/a | | |
| **Cisgender women** | | |  |  |  |  |  |  |
| **Uptake of HIV Testing^a^** | | |  |  |  |  |  |  |
|  | **Intervention effects at 8 months** | | |  |  |  |  |  |
|  |  | HIVST vs SOC | 113.39 | 33.13, 388.03 | <0.001 | 244.8 | 52.82, 1134.55 | <0.001 |
|  |  | HIVST+mHealth vs SOC | 67.10 | 21.08, 213.59 | <0.001 | 161.41 | 38.11, 683.64 | <0.001 |
|  | **Intervention effects at 12 months** | | |  |  |  |  |  |
|  |  | HIVST vs SOC | 209.85 | 22.17, 1986.7 | <0.001 | 460.60 | 36.68, 5784.2 | <0.001 |
|  |  | HIVST+mHealth vs SOC | 38.93 | 9.56, 158.55 | <0001 | 77.11 | 15.20, 391.11 | <0.001 |
| **HIV Status Knowledge^b^** | | |  |  |  |  |  |  |
|  | **Intervention effects at 12 months** | | |  |  |  |  |  |
|  |  | SOC | ref | | |  |  |  |
|  |  | HIVST & HIVST + mHealth | 66.83 | 8.54, 523.05 | <0.001 | 142.98 | 9.48, 2156.45 | <0.001 |
| **Unused HIVST Kits^c^** | | |  |  |  |  |  |  |
|  | **Intervention effects at 16 months follow-up** | | |  |  |  |  |  |
|  |  | HIVST | ref | | | ref | | |
|  |  | HIVST + mHealth | 9.94 | 2.73, 36.20 | <0.001 | 6.56 | 0.88, 48.83 | 0.066 |
| **Note:** OR, odds ratio; CI, confidence interval; HIVST, HIV self-test; SOC, standard of care. ^a^Intervention effect on uptake of HIV testing is estimated as the interaction between intervention arm and time point, calculated using generalized estimating equation logistic regression models with an unstructured correlation matrix. ^b^Intervention effect on HIV status knowledge is only measured at 12 months for both intervention arms compared to SOC, calculated using logistic regression models. ^c^Intervention effect on use of HIVST kits is measured at 16 months follow-up for HIVST+mHealth arm compared to HIVST arm, calculated using logistic regression models. *Adjusted for pre-specified covariates (age, gender) and baseline imbalances (birth country, employment, income security, relationship status) | | | | | | | | |

**Supplemental Table 4.** Retrospective assessment of Tushirikiane study pragmatism using PRECIS-2

|  | **Domain** | **Investigator 1 PRECIS-2 score** | **Investigator 2 PRECIS-2 score** | **Investigator 2 PRECIS-2 score** | **Median score** | **Rationale** |
| --- | --- | --- | --- | --- | --- | --- |
| 1 | Eligibility criteria: *Who is selected to participate in the trial?* | 4 | 5 | 5 | 5 | all refugee youth were eligible; no need to meet HIV risk profile; criteria was broad for inclusion; most refugees in Kampala own a phone |
| 2 | Recruitment path: *How are participants recruited into the trial?* | 2 | 3 | 3 | 3 | there was a lot of recruitment effort by peers navigators and community partners, but no formal advertisement |
| 3 | Setting: *Where is the trial being done?* | 4 | 5 | 5 | 5 | -trial was largely conducted in participant homes in slum and informal settlements; community collaborator also located in informal settlement close to real world (not a clinical setting) |
| 4 | Organization intervention: *What expertise and resources are needed to deliver the intervention?* | 4 | 4 | 4 | 4 | -we trained refugee youth and non-experts (peer navigators who were also refugee youth) in the delivery of the intervention; used largely the resources available at the refugee community agency located in an informal settlement |
| 5 | Flexibility of intervention (delivery): *How should the intervention be delivered?* | 2 | 3 | 3 | 3 | -in usual care, youth would have to go to a clinic or pharmacy to buy HIV self-test kits whereas we provided the kits to them; the clinics the participants received confirmatory tests at were close to their homes |
| 6 | Flexibility of intervention (adherence): *What measures are in place to make sure participants adhere to the intervention?* | 2 | 2 | 2 | 2 | -we provided weekly check-ins with bidirectional SMS to ensure participants had support as needed to take part in the intervention |
| 7 | Follow up: *How closely are participants followed-up?* | 2 | 2 | 2 | 2 | - peer navigators followed up closely with participants by multiple means, including phone, SMS, and WhatsApp |
| 8 | Outcome: *How relevant is it to participants?* | 5 | 5 | 5 | 5 | -HIV status knowledge is an important outcome in high prevalence context |
| 9 | Analysis (To what extent are all data included? | 5 | 5 | 5 | 5 | -all data is included in analyses |

**Note:** PRECIS-2 Scoring guide: 1) very explanatory (ideal conditions), 2) rather explanatory, 3) equally pragmatic and explanatory, 4) rather pragmatic, 5) Very pragmatic (closest to real world conditions)
